# Intermittent Hypoxia and Respiratory Plasticity: The Good, the Bad, and the Unknown

**DOI:** 10.1093/function/zqad045

**Published:** 2023-09-04

**Authors:** Andrew William Sheel

**Affiliations:** School of Kinesiology, The University of British Columbia, British Columbia V6T 1Z3, Vancouver, Canada

## A Perspective on “APOE4, Age, and Sex Regulate Respiratory Plasticity Elicited by Acute Intermittent Hypercapnic-Hypoxia”

What are the effects of exposure to hypoxia in humans and other mammalian species? The question is purposely vague as the answer will inevitably be: *it depends*. Consider the hypoxia of high altitude. On one hand, approximately 80 million people reside >2500 m above sea level and select populations (ie, Andean, Tibetan, Ethiopian) have lived at high altitude for millennia. There is now a growing appreciation that they appear to have acquired distinctive physiological phenotypes in order to live in their respective hypoxic environments. On the other hand, when sea-level residents travel to high altitude, a host of physiological responses takes place that are critical for successful acclimatization with significant between-person variation. The question becomes more complex with increasing levels of high altitude and is further complicated with the physiological stressor of exercise (ie, the suite of physiological changes necessary to match O_2_ delivery to metabolic demand). Consider the sea-level mountain climber who wishes to ascend to >5000 m in a relatively short amount of time without the benefit of an ancestral high-altitude phenotype. Remarkably, these feats can be accomplished without supplemental O_2_, but there is also a list of unfortunate events where mountaineers have succumbed to the harsh conditions of severe high-altitude hypoxia. This final point serves to underscore that there are limits to the human tolerance of hypoxia and it is an insult to O_2_ transport.

Clinically, hypoxia can accompany several pathophysiological states. For example, in the field of sleep medicine, hypoxia is intermittent in nature with respect to sleep apnea. Obstructive sleep apnea (OSA) is a chronic medical disorder of the respiratory system during which patients experience intermittent hypoxia (IH), often for many years prior to diagnosis and treatment. The chronic IH exposure is thought to be responsible for many of the long-term cardiovascular consequences associated with OSA, including systemic hypertension, myocardial infarction, and stroke. Epidemiologic studies have highlighted these associations, yet our understanding of the underlying mechanisms is incomplete. Experimental animal studies have demonstrated that exposure to severe IH is accompanied by sustained increases in blood pressure that are linked to chronic increases in sympathetic nervous system activity.^1^ Human studies that have sought to eliminate IH with continuous positive airway pressure show that while hypoxemia may contribute to increases in blood pressure, the effect is small or absent in the case of mild to moderate hypoxemia.^2^ Additional work is required to fully understand the link between IH, enhanced sympathetic nervous system activity, and hypertension.

As pointed out by Dempsey and Morgan,^3^ the responses to hypoxia can be adaptive and maladaptive. While IH is typically viewed as a high-risk stimulus with respect to patient populations, there is a growing appreciation that some types of experimentally imposed IH of low frequency, moderate severity, and short duration can elicit possible clinical benefits. For example, short-term exposures to IH (via manipulation of the fraction of inspired O_2_) alternating with normoxia in both rodents and humans have shown respiratory motoneuron with enhancement of ventilation in rodents and humans with spinal cord injury.^4,5^ Furthermore, IH increases motor function in humans with incomplete spinal cord injury.^6^ Our comprehension of the effects of IH continues to grow and the recent study of Nair et al.^7^ adds important new insight into our understanding. With a carefully designed set of experiments, they showed that acute exposure to IH induces respiratory plasticity in healthy humans and this can differ on the basis of genetics, age, and sex. Here, healthy humans were exposed to IH (9.5% O_2_) or sham (room air) conditions. Measured variables included diaphragm motor-evoked potentials (MEPs), respiratory drive, mouth occlusion pressure, and several single-nucleotide polymorphisms in genes known to be associated with phrenic motor plasticity and neuronal plasticity (apolipoprotein E [APOE]). Impressively, the human study was accompanied by humanized APOE knock-in rat experiments. Key findings include the following. First, diaphragm MEP following IH were higher following IH and were lower in people heterozygous for the APOE4 allele. Second, males had a greater change in diaphragm MEPs than females following IH and this did not differ on the basis of age. Third, there was a negative association between age and change to respiratory drive. Lastly, the human experiments in knock-in rats indicate a causal relationship between apolipoprotein genotype and impaired respiratory motor plasticity. Collectively, the findings provide strong evidence that the APOE4 allele, age, and sex are key determinants of human respiratory motor plasticity.

The biological significance of the study is the identification of a mechanistic basis for the persistent change in the neural control of breathing. The authors have been careful to not overinterpret their findings and translate the results to patient groups. With this in mind, the study serves as an exciting and important start point for the design of future studies addressing the possible therapeutic value of IH to preserve or restore breathing function in people with neurological disorders, such as spinal cord injury and amyotrophic lateral sclerosis. The research participants (*n* = 17) in this study included healthy males and females across a range of ages (20 to 40 yr). The study design does not necessarily speak to the idea that different environmental factors may (or may not) have direct or indirect epigenetic changes that modify the effects of IH. A number of questions need to be addressed before the effects of IH across different groups of humans are fully understood. For example, the authors make the point that the menstrual cycle phase in female participants was not controlled for in this study and that menstrual cycle-related effects on neuroplasticity may be influenced by a number of factors. Moreover, might the effects of IH be different pre- and post-menopause? In addition, healthy aging is known to affect structural and functional components of the respiratory system. As such, to fully understand the effects of IH on respiratory plasticity, epigenetic factors across the human lifespan require careful attention and consideration. The results of Nair et al.^7^ serve as an exemplar of carefully conceived and well-executed studies that provide a solid base on which others in the field can stand.
